# Extracorporeal Shock Wave Therapy (ESWT) in burn scar microbiome modulation: critical analysis of methodological rigor and translational potential

**DOI:** 10.1097/JS9.0000000000002411

**Published:** 2025-04-18

**Authors:** Bo Wang, Xiaohong Zheng, Xiaojuan Zhang

**Affiliations:** a The Affiliated Bishan Hospital of Chongqing Medical University, Chongqing, China; b Chongqing Medical and Pharmaceutical College, Chongqing, China

*Dear Editor,*

We have read the research by Jung *et al* with great interest^[^1^]^. This study provides a novel investigation into the effects of extracorporeal shock wave therapy (ESWT) on the skin microbiome of burn scars. The study’s principal contribution lies in its unique focus on a relatively under-researched area, filling a significant gap in the existing literature regarding the relationship between ESWT and the skin microbiome in burn patients. While the methodological design demonstrates rigor, several aspects warrant further scrutiny.

First, from a methodological perspective, the exclusion criteria explicitly ruled out patients with pre-existing skin diseases (e.g., dermatitis, psoriasis), metabolic disorders (e.g., diabetes), and those using antibiotics. However, the study failed to report whether participants had used probiotics or followed dietary regimens that might influence microbial communities. Dietary habits and probiotic intake are critical modifiers of the skin microbiome, and their omission weakens the ability to isolate ESWT-specific effects^[^2,3^]^. Additionally, the protocol did not specify whether participants developing skin/systemic infections, febrile illnesses, or other disorders affecting the skin microbiome were excluded during the 3-month period. Such conditions could confound microbiome results, yet their absence in exclusion criteria introduces a potential bias.

The reliance on 16S rRNA sequencing, while cost-effective for genus-level analysis, restricts species- or strain-level resolution, potentially overlooking critical functional differences between closely related taxa (e.g., pathogenic vs. commensal Staphylococcus species). Furthermore, 16S data lack functional insights into microbial metabolism or host-microbe interactions, limiting mechanistic interpretations of observed diversity changes.

Second, sampling at the 3-month mark (3M) was conducted immediately post-treatment, aligning with the final ESWT session. While this timing captures acute post-intervention changes, the lack of long-term follow-up (e.g., 6 or 12 months) limits insights into the durability of observed microbiome alterations.

Third, this study documented significant reductions in pain and itching scores after Extracorporeal Shock Wave Therapy (ESWT), but omitted the detailed reporting of treatment-related side effects (such as pain, erythema, edema, or skin irritation)^[^4,5^]^. Such data are essential for evaluating the safety profile of ESWT in clinical practice. At the same time, these data are also important factors influencing the skin microbiome and some blood chemistry.

Overall, the findings highlight increased alpha diversity and shifts toward commensal genera (e.g., *Staphylococcus* and *Micrococcus*) in treated scars, suggesting ESWT fosters a healthier microbial environment. Network analysis revealing a more open microbial structure further supports its potential role in scar remodeling. However, the small sample size (*n* = 19) and retrospective design limit generalizability. The conclusion that ESWT promotes a “balanced” microbiome is compelling but requires validation through larger randomized controlled trials (RCTs) with longitudinal follow-up. The reliance on 16S sequencing introduces uncertainty about whether observed taxonomic shifts translate to functionally meaningful changes. Translational implications require validation through larger, randomized controlled trials integrated with metagenomic or metatranscriptomic approaches to resolve species/strain-level variations and functional profiles.

## Data Availability

Not applicable.
